# Mental Pain Surrounding Suicidal Behaviour: A Review of What Has Been Described and Clinical Recommendations for Help

**DOI:** 10.3389/fpsyt.2021.750651

**Published:** 2022-01-27

**Authors:** Susana Morales, Jorge Barros

**Affiliations:** ^1^Psychiatry Department, School of Medicine, Pontificia Universidad Católica de Chile, Santiago, Chile; ^2^Millennium Institute for Research in Depression and Personality (MIDAP), Santiago, Chile

**Keywords:** suicide prevention, suicidal mental pain, suicide intervention, suicide clinical assessment, suicidal states and traits

## Abstract

**Objective:**

To conduct a comprehensive review of scientific publications related to mental pain and suicide risk in order to deepen relevant aspects to guide clinical interventions.

**Method:**

Using a text analysis tool, we collected the terms most frequently linked with that situation in published results of research using various tools to evaluate mental pain or psychache.

**Discussion:**

We propose clinical interventions for the clinical conditions most commonly associated with mental pain.

## Introduction

For decades, suicide and its associated behaviours (SB) have been considered as priority problems in the health policies of many countries and the world (78). Despite this, we do not have adequate strategies to significantly reduce its incidence. This is explained by the complex nature of this behaviour, in which sociocultural, familial, biological, and psychological aspects concur. This is what many studies conducted in different countries over several decades consistently teach (1).

Today, despite our solid knowledge of the different variables associated with this behaviour, it has not been possible to generate interventions that allow us to anticipate SB (2). This is a limitation that includes the estimation of immediate and future behaviour. Given this, many authors have raised the need to study this behaviour using methods other than traditional ones (3, 4), in what some have described as a “paradigm shift.” An example of the limitations that traditional SB risk indicators face in order to anticipate these behaviours can be seen in studies that have explored the clinical utility of SB severity ranking scales. The available evidence does not support the clinical use of these scales (5, 6). Furthermore, there is considerable agreement regarding the uselessness and eventual risk of the use of scales and other clinical instruments in order to rank suicide risk in risk subjects (7–9).

SB is infrequent even in those patients whose clinical condition is frequently associated with risk factors. An example of this is the case of having a previous history of suicide attempts. While a prior suicide attempt is one of the best predictors of suicide risk, most of those who attempt suicide will die from another cause. This shows the importance of state variables in the risk of SB. When comparing depressive patients with and without SB, we can recognise a set of variables that, when presented simultaneously, could configure a risk state for SB (10). But we can also describe what the psychic experience that precedes the suicidal behaviour is like in order to identify a particular clinical condition in those who might have SB. The latter is what Shneidman proposed when underlining the crucial role that a specific psychic state has as the antecedent of all SB: “in most cases, suicide stems from psychological pain, and the psychache itself arises from frustrated psychological needs” (11). For this important author, all SB was preceded by a state that he defined as “psychache” (12). This term describes a state of intense disturbance that has also been defined as “mental pain” (MP), “psychological pain,” and “psychic pain” by different authors (13). For many, this state represents a way of responding to adversity and suffering that seems inevitable, and that is what would incline someone to end his/her life.

The role of psychache as a mediator between circumstances, risk factors, and suicidal behaviour has led several authors to recognise the central role that psychache plays in SB (14). Several scales have been developed to recognise and identify its severity (13, 15, 16). One of the most recent proposals is the incorporation of this dimension as part of the patient-reported outcomes (PRO) in treatment outcome studies (15). But there are two difficulties in this: firstly, the capacity in those who experience this state to understand it as MP, and secondly, the difficulty for clinicians to interpret the different aspects of the mental examination under the concept of MP.

Indeed, a particular problem of SB is the knowledge that the patient has of his/her own state. It is quite possible that psychache's own deep emotional distress and disturbance prevents many subjects from being able to understand their behaviour and decisions. For some, the latter forces patients to consider the possibility of suicidal behaviour, even if they do not express it explicitly (17). If this is the case, then those who evaluate patients with severe psychiatric problems should assess the general condition of the patient without restricting themselves exclusively to what the patient says when assessing the risk of SB. This clinical problem would allow us to understand those cases of subjects who consulted for a short time before committing suicide without manifesting ideation or death plans when being evaluated. In the latter cases, it is often thought that the subjects concealed their will, and although in many cases this could have been the case, it is possible that many of them were unaware of the state of risk in which they were.

Today we have several scales to assess the severity of MP and psychache that have been used in samples of very diverse groups of patients at risk of suicide (adolescents, students, the elderly, migrants, patients with a history of high-risk suicide attempts). As some point out, “mental pain provides the clinical threshold that is essential for determining the amount of distress that is worthy of clinical attention, in conjunction with diagnostic criteria. It may be a better specification of the criteria on ‘clinically significant distress' that frequently recurs in DSM IV” (13). On the other hand, this state would be a factor associated with suicide independent of the diagnosis. MP can be established progressively during the different stages of suicidal behaviour—ideation, attempt, and suicide—as a continuous phenomenon. The association of other factors with the state of MP could increase or decrease its intensity. Although these scales help to assess the severity of MP, it is not enough to recognise its existence and severity, since it is necessary to know for each subject what factors explain this state in her/him. The latter is what a good clinical interview should achieve by identifying what facts and factors promote this state in each individual subject (13). Research on suicidal behaviour has always tried to find what might prevent it. For some, the task should be broader, since the research should also be directed to identify aspects that could be a target for intervention: “Existing suicide prediction models which attempt to pinpoint the patients at highest suicide risk, do not tell us which suicide-focused treatments are right for which patient. This is the key clinical question critics of suicide risk screening have in mind when they say in-depth clinical assessments should focus on need for treatment rather than risk of suicide” (7). By establishing the determining factors of MP for each patient, we could then define interventions to mitigate the impact of this state according to highly individual needs.

The aim of this study is to contribute to the accuracy of the understanding of MP in suicide risk through a qualitative review of 73 studies that present findings about the state and experience associated with MP in suicide risk. This information will help clinicians to guide the clinical interview to assess those areas of psychic life typically associated with MP. For this purpose, we have selected studies conducted to evaluate MP or Psychache following the same criteria used by the authors of a recent major review (14). Our findings seek to translate the information from many studies carried out in diverse populations into the clinic.

MP is a transdiagnostic construct which has been recognised as one of the most important proximal predictors of suicidal risk well beyond depression (14, 18, 19). “MP is also understood as “psychache” (20) and defined as an acute state of intense psychological pain associated with negative cognitive and emotional aspects of the self—i.e., thoughts of self-disappointment or inadequacies and feelings of guilt, anguish, fear, panic, angst, loneliness, and helplessness often accompanied by a sense of disconnection, loss, or incompleteness of the self (13, 21, 22).

Aspects that stand out in suicidal MPs are certain experiences of connectedness and impulsivity. On the one hand, connectedness would be associated with: feeling socially excluded; loneliness-even when apparently accompanied by others; not having mutual and satisfactory interpersonal relationships; perception of being a burden to others. This is linked [according to the Interpersonal Theory of Suicide proposed by Joiner (23)] to an acquired capacity to commit suicide. Impulsivity, on the other hand, MP would be related to the experience of out-of-control expressions of aggression, trait anger, risk-taking behaviours in the face of intolerable and impossible to communicate experiences in requesting help (24).

With regard to the above, a development of clinical interventions aimed at addressing connectedness and the management of reactive impulsivity would be especially beneficial for clinical interventions. This would be particularly useful in critical services such as psychiatric emergencies and in the case of pathologies usually associated with suicidal risk, such as depressive disorders, dependencies, psychotic disorders, eating disorders and personality dysfunctionalities (25, 26).

MP is viewed as stemming from the discrepancy between the ideal and the actual perception of oneself, accompanied by the awareness of one's role in the experience of emotional pain (13). This suffering would be found around a perception of the gap between what we want and what we can have. Along with the realisation of the existence of this gap, there is a certain impossibility of tolerating this gap. MP sufferers accept that the gap exists and are unable to intuit alternatives to find solutions besides death.

We agree with the literature demonstrating that the moment bordering on suicidal ideation and attempt is affected by a particular emotional transitory state and a personality style characterised by traits that lead to the activation of suicidal risk.

A psychological state is a construct defined as characteristic patterns of thinking, feeling, and behaving in a concrete situation at a specific moment in time; it is a transitory experience that can be affected by external events and by internal movements of the self. State variables such as depression, hopelessness, and anxiety have been shown to have a significant relationship with suicide risk (27).

Other factors include stable or enduring differences between individuals. Such long-term tendencies, sometimes referred to as traits, could be a wistful temperament, genetic predisposition, personality style (27), or characteristic patterns or tendencies of thinking, feeling, and behaving that generalise across similar situations, differ systematically between individuals, and remain rather stable across time (28). Self-destructiveness, poor impulse control, and antisocial behaviour are among the main suicide risk factors observed in suicidal patients with personality dysfunctions (29).

Our purpose through this comprehensive study is to pinpoint those factors most commonly associated with suicide risk, as shown by studies examining MP and suicide. Using this information, we propose clinical interventions that would allow us to reduce the state of MP.

## Materials and Methods

We carried out a comprehensive review of scientific publications that studied the concept of mental pain, offering a high-quality data synthesis of the studies, according to the following search criteria:

- Systematic reviews and original research from 1996 to 2021 that were published in international scientific journals in the English language, describing mental pain and psychache in relation to suicidal ideation, suicide attempts, suicidal behaviour, and high-lethality suicide attempts.
- Among the exclusion criteria, we considered meta-analyses, letters to the editor, single-case studies, general reviews, conference presentations, books, and abstracts.
- The search was directed to the following platforms: Web of Science, PubMed, Scopus, Google Scholar, PsycLit, and ProQuest.
- The search keywords were psychological suffering OR psychache OR emotional pain OR psychic pain OR psychological pain OR mental pain OR self-harm AND suicid^*^.

This inquiry yielded 73 studies. The details are shown in Table 1. The heterogeneity of the resulting scientific articles in terms of methods and sample types made it impossible to gather criteria for quantitative analysis. Given the above, we performed a qualitative analysis in three successive steps:

1. Qualitative review of concepts associated with mental pain and psychache in suicidal crisis, based on the results and discussion sections of the reviewed articles.
2. For first-word filtering, the text mining package (tm) library for R was used.
3. The concepts that stood out for their repetition were selected, for which we used a free program available online which organises texts according to their content and frequency of appearance—Text Analyzer (https://www.online-utility.org/text/analyzer.jsp). Points, spaces, prepositions, and conjunctions were filtered.

**Table 1:** Detail of papers in comprehensive review of scientific publications that studied the concept of mental pain.

	**Reviewed papers**	**Year**	**Type of study**	**Sample**
1	Barak A, Miron O. Writing characteristics of suicidal people on the internet: a psychological investigation of emerging social environments. Suicide Life Threat Behav (2005) 35(5):507–24. doi: 10.1521/suli.2005.35.5.507	2005	Quantitative	3 samples: 65, 80, 63 sujects
2	Becker, G., Orbach, I., Mikulincer, M., Iohan, M., Gilboa-Schechtman, E., & Grossman-Giron, A. (2019). Reexamining the mental pain–suicidality link in adolescence: The role of tolerance for mental pain. Suicide and Life Threating Behavior, 49(4), 1072–1084. doi: https://doi.org/10.1111/sltb.12506	2019	Quantitative	Study 1: 183 nonclinical sample Study 2: 139 clinical sample Study 3:24 psychiatric patients with suicidality, 24 non-suicidal psychiatric patients, 24 adolescents non-clinical,
3	Bolger, E. (1999). Grounded theory analysis of emotional pain. Psychotherapy Research, 9(3), 342–362. doi: https://doi.org/10.1080/10503309912331332801	1999	Qualitative	7 adults
4	Cáceda, R., Durand, D., Cortes, E., Prendes-Alvarez, S., Moskovciak, T., Harvey, P. D., & Nemeroff, C. B. (2014). Impulsive Choice and Psychological Pain in Acutely Suicidal Depressed Patients. Psychosomatic Medicine, 76(6), 445–451. doi: 10.1097/psy.0000000000000075 (2014) 44(1):77–88. doi: 10.111/sltb.12056	2014	Quantitative	82 adults 18–65.
5	Campos R, Holden R, Santos S. (2018). Exposure to suicide in the family: Suicide risk and psychache in individuals who have lost a family member by suicide. J. Clin. Psychol. 2018;74:407–417. doi: 10.1002/jclp.22518	2018	Quantitative	225 subjects, 53 patients, and 172 controls
6	Campos RC, Holden RR, Laranjeira P, Troister T, Oliveira AR, Costa F, et al. Self-report depressive symptoms do not directly predict suicidality in nonclinical individuals: contributions toward a more psychosocial approach to suicide risk. Death Stud (2016) 40(6):335–49. doi: 10.1080/07481187.2016.1150920	2016	Quantitative	961 subjects for 3 different samples
7	Campos RC, Holden RR. Testing models relating rejection, depression, interpersonal needs, and psychache to suicide risk in nonclinical individuals. J Clin Psychol (2015) 71(10):994–1003. doi: 10.1002/jclp.22196	2015	Quantitative	203 adults (100 males, 103 women)
8	Coohey C, Easton S, Kong J, Bockenstedt JKW. Sources of psychological pain and suicidal thoughts among homeless adults. Suicide Life Threat Behav (2015) 45(3):271–80. doi: 10.1111/sltb.12126	2015	Quantitative	457 subjects
9	DeLisle M, Holden RR. Differentiating between depression, hopelessness, and psychache in university undergraduates. Meas Eval Couns Dev (2009) 42(1):46–63. doi: 10.1177/0748175609333562	2009	Quantitative	587 students undergraduated
10	Demirkol, M.E., Tamam, L., Naml, Z., Eriş Davul, Ö., 2019. Validity and reliability study of the Turkish version of the tolerance for mental pain scale-10. Psychiat. Clin. Psych.	2019	Quantitative	121, 62 with SB
11	Esther L. Meerwijk and Sandra J. Weiss, Tolerance for Psychological Pain and Capability for Suicide: Contributions to Suicidal Ideation and Behavior, Psychiatry Research, doi: https://doi.org/10.1016/j.psychres.2018.02.005	2018	Quantitative	219 facebook users
12	Farshid Shamsaei, Safura Yaghmaei & Mohammad Haghighi (2020) Exploring the lived experiences of the suicideattempt survivors: a phenomenological approach, International Journal of Qualitative Studies on Health and Well-being, 15:1, 1745478. doi: 10.1080/17482631.2020.1745478	2020	Qualitative	16 subjects, in depth interviews
13	Fertuck, E. A., Karan, E., & Stanley, B. (2016). The specificity of mental pain in borderline personality disorder compared to depressive disorders and healthy controls. Borderline Personality Disorder and Emotion Dysregulation, 3(2), 1–8. doi: https://doi.org/10.1186/s40479-016-0036-2.	2016	Quantitative	79 patients and 31 controls
14	Flamenbaum R, Holden RR. Psychache as a mediator in the relationship between perfectionism and suicidality. J Couns Psychol (2007) 54(1):51–61. doi: 10.1037/0022-0167.54.1.51	2007	Quantitative	264 undergraduate students
15	Fleming, M. (2006). Distinction between mental pain and psychic suffering as separate entities in the patient's experience. International Forum of Psychoanalysis, 15(4), 195–200. doi: https://doi.org/10.1080/08037060500522754.	2006	Qualitative	Theory review
16	Gould MS, Kalafat J, HarrisMunfakh JL, Kleinman M. An evaluation of crisis hotline outcomes part 2: suicidal callers. Suicide Life Threat Behav (2007) 37(3):338–52. doi: 10.1521/suli.2007.37.3.338	2007	Quantitative	1.085 patients with SB evaluated, and 380 (35.0%) followed up.
17	Grossman-Giron, A., Becker, G., Kivity, Y., Shalev, S., & Tzur Bitan, D. (2020). Mental pain intensity and tolerance as predictors of psychotherapy process and outcome. Journal of Clinical Psychology. doi: https://doi.org/10.1002/jclp.23085	2020	Quantitative	53 Patients
18	Guimarães, R., Fleming, M., & Cardoso, M. F. (2014). Validation of the Orbach & Mikulincer Mental Pain Scale (OMMP) on a drug addicted population. Social Psychiatry and Psychiatric Epidemiology, 49(3), 405–415. doi: https://doi.org/10.1007/s00127-013-0751-6.	2014	Quantitative	403 patients
19	Gvion Y, Levi-Belz Y. (2018). Serious Suicide attempts: Systematic Review of Psychological Risk Factors. Frontiers in Psychiatry doi: 10.3389/fpsyt.2018.00056	2018	Systematic review	39 studies
20	Gvion, Y., Horesh, N., Levi-Belz, Y., Fischel, T., Treves, I., Weiser, M., et al. (2014). Aggression-impulsivity, mental pain, and communication difficulties in medically serious and medically non-serious suicide attempters. Comprehensive Psychiatry, 55(1), 40–50. doi: https://doi.org/10.1016/j.comppsych.2013.09.003.	2014	Quantitative	196 subjects
21	Gviona Y, Horesh N, Levi-Belz Y, Apter A. A proposed model of the development of suicidal ideations. Compr Psychiatry (2015) 56:93–102. doi: 10.1016/j.comppsych.2014.09.019	2015	Quantitative	196 subjects.
22	Holden RR, Mehta K, Cunningham EJ, McLeod LD. Development and preliminary validation of a scale of psychache. Can J Behav Sci (2001) 33:224–32. doi: 10.1037/h0087144	2001	Quantitative	Study 1: 294 age average promedio de edad 19,1 (sd: 1,6) 102 subjects: Study 2: 211 women (19,4 sd = 2,4)
23	Horesh N, Levi Y, Apter A. Medically serious versus non serious suicide attempts: relationships of lethality and intent to clinical and interpersonal characteristics. J Affect Disord (2012) 136:286–93. doi: 10.1016/j.jad.2011.11.035	2012	Quantitative	102 subjects:
24	Huanhuan L, Weizhen X, Xinwei L, Rog F, Chuan S, Xiangyu Y, et al. Clarifyng the role of psychological pain in the risks of suicidal ideation and suicidal acts among patients with major depressive episodes. Suicide Life Threat Behav (2014) 44(1):77–88. doi: 10.111/sltb.12056	2014	Quantitative	111 patients
27	Ismael Conejero, Emilie Olié, Raffaella Calati, Déborah Ducasse, Philippe Courtet (2018). Psychological Pain, Depression, and Suicide: recent evidence and Future Directions. Current Psychiatry Reports (2018) 20: 33	2018	Theory review	Theory review
25	Jollant F, Near J, Turecki G, Richard-Devantoy S. (2016). Spectroscopy markers of suicidal risk and mental pain in depressed patients. Progress in Neuropsychopharmacology & Biological Psychiatry doi: 10.1016/j.pnpbp.2016.10.005	2016	Quantitative	25 pacients and 33 controls.
26	Lambert C, Troister T, Ramadan Z, Montemarano V, Fekken C, Holden R. (2020). Psychache Predicts Suicide Attempter Status Change in Students Starting University. Suicide and Life-Threatening Behavior 50 (3) June 2020 © 2020 The American Association of Suicidology doi: 10.1111/sltb.12624	2020	Quantitative	516 university students follow up, 10 weeks
27	Landi, G., Furlani, A., Boccolini, G., Mikulincer, M., Grandi, S., & Tossani, E. (2020). Tolerance for Mental Pain Scale (TMPS): Italian validation and evaluation of its protective role in depression and suicidal ideation. Psychiatry Research, 291, 113263.	2020	Quantitative	204 participantes entre 18 y 68
28	Leenars AA, Lester D. A note on Shneidman's psychological pain assessment scale. Omega: J Death Dying (2005) 50(4):301–7. doi: 10.2190/WH9X-80M3-NJ54-5GCU	2005	Quantitative	127 subjects: 37 males 90 female (22,9 sd 6,4)
29	Lester D. Psychache, depression, and personality. Psychol Rep (2000) 87:940. doi: 10.2466/PR0.87.7.940-940	2000	Quantitative	51 students
30	Levi Y, Horesh N, Fischel T, Treves I, Or E, Apter A. Mental pain and its communication in medically serious suicide attempts: an “impossible situation”. J Affect Disord (2008) 111:244–50. doi: 10.1016/j.jad.2008.02.022	2008	Quantitative	102 patients with SB, 71 controls
31	Levi-Belz Y, Gvion Y, Grisaru S & Apter A. (2018) When the Pain Becomes Unbearable: Case-Control Study of Mental Pain Characteristics Among Medically Serious Suicide Attempters, Archives of Suicide Research, 22:3, 380–393. doi: 10.1080/13811118.2017.1355288	2018	Quantitative	241 participants (142 men, 99 women) 20–85 years old
32	Levi-Belz Y, Gvion Y, Horesh N, Apter A. Attachment patterns in medically serious suicide attempts: the mediating role of self-disclosure and loneliness. Suicide Life Threat Behav (2013) 43(5):511–22. doi: 10.1111/sltb	2013	Quantitative	102 subjects
33	Levinger, S., Somer, E., & Holden, R. R. (2015). The importance of mental pain and physical dissociation in youth suicidality. Journal of Trauma & Dissociation, 16(3), 322–339. doi: https://doi.org/10.1080/15299732.2014.989644	2015	Quantitative	123 subjects
34	Li, S., Yaseen, Z. S., Kim, H. J., Briggs, J., Duffy, M., Frechette-Hagan, A., Cohen, L. J., & Galynker, I. I. (2018). Entrapment as a mediator of suicide crises. BMC psychiatry, 18(1), 4. doi: https://doi.org/10.1186/s12888-018-1587-0	2018	Quantitative	200 subjects 18–65 years old
35	Yang, L., Liu, X., Chen, W., & Li, L. (2019). A Test of the Three-Step Theory of Suicide among Chinese People: A Study Based on the Ideation-to-Action Framework. Archives of suicide research: official journal of the International Academy for Suicide Research, 23(4), 648–661. doi: https://doi.org/10.1080/13811118.2018.1497563	2019	Quantitative	1,097 subject non-clinical 594 women, three public universities in China
36	Mee S, Bunney BG, Bunney WE, Hetrick W, Potkin SG, Reist C. Assessment of psychological pain in major depressive episodes. J Psychiatr Res (2011) 45:1504–10. doi: 10.1016/j.jpsychires.2011.06.011	2011	Quantitative	73 subjects, 96 control (non-psyquiatrics)
37	Mee, S., Bunney, B. G., Reist, C., Potkin, S. G., & Bunney, W. E. (2006). Psychological pain: A review of evidence. Journal of Psychiatric Research, 40(8), 680–690. doi: https://doi.org/10.1016/j.jpsychires. 2006.03.003.	2006	Review	
38	Meerwijk, E. L., & Weiss, S. J. (2011). Toward a unifying definition of psychological pain. Journal of Loss and Trauma, 16(5), 402–412. doi: https://doi.org/10.1080/15325024.2011.572044.	2011	Revisión Sistemática	6 studies
39	Meerwijk, E. L., Chesla, C. A., & Weiss, S. J. (2014). Psychological pain and reduced resting state heart rate variability in adults with a history of depression. Psychophysiology, 51(3), 247–256. doi: https://doi.org/10.1111/psyp.12175.	2014	Quantitative	35 depresive adults
40	Meerwijk, E. L., Mikulincer, M., & Weiss, S. J. (2019). Psychometric evaluation of the tolerance for mental pain scale in United States adults. Psychiatry Research, 273, 746–752. doi: https://doi.org/10.1016/j.psychres.2019.01.101.	2019	Quantitative	225 Facebook users
41	Meerwijk, E.L., Ford, J.M., Weiss, S.J., 2013. Suicidal crises because of diminishing tolerance to psychological pain. Brain Imag. Behav. 7, 245–247. doi: https://doi.org/10.1007/s11682-012-9179-y.	2013	Teoric reviex	Teoric review
42	Mills J, Green K, Reddon J. An evaluation of the psychache scale on an offender population. Suicide Life Threat Behav (2005) 35(5):570–80. doi: 10.1521/suli.2005.35.5.570.	2005	Quantitative	136 men imprisoned by the law
43	Montemarano V, Troister T, Lambert C, Holden R. (2018). A four-year longitudinal study examining psychache and suicide ideation in elevated-risk undergraduates: A test of Shneidman's model of suicidal behavior. J. Clin. Psychol. 2018;74:1820–1832. doi: 10.1002/jclp.22639.	2018	Quantitative	82 high suicidal university students
44	Ohana, I., Golander, H., & Barak, Y. (2014). Balancing psychache and resilience in aging holocaust survivors. International Psychogeriatrics, 26, 929–934.	2014	Quantitative	214 participants; 101 women, 113 men
45	Olié E, Guillaume S, Jaussent I, Courtet P, Jollant F. Higher psychological pain during a major depressive episode may be a factor of vulnerability to suicidal ideation and act. J Affect Disord (2010) 120:226–30. doi: 10.1016/j.jad.2009.03.013.	2010	Quantitative	210 inpatients hospitalised with major depression diagnosis
46	Orbach, I., Mikulincer, M., Gilboa-Schechtman, E., & Sirota, P. (2003). Mental pain and its relationship to suicidality and life meaning. Suicide and Life-Threatening Behavior, 33(3), 231–241. doi: https://doi.org/10.1521/suli.33.3.219.23219.	2003	Quantitative	Study 1: 32 adults 14 men y 18 women Study 2: 98 israel students 79 women 23 men
47	Orbach, I., Mikulincer, M., Sirota, P., & Gilboa-Schechtman, E. (2003). Mental pain: a multidimensional operationalization and definition. Suicide and Life-threatening Behavior, 33(3), 219–230. doi: 10.1521/suli.33.3.219.23219.	2003	Mixed	Study 2: 255 israel students (194 women 61 men) Study 3: 100 students
48	Pachkowski M, May A, Tsai M, Klonsky, D. (2019). A Brief Measure of Unbearable Psychache. Suicide and Life-Threatening Behavior 49 (6) December 2019 1721© 2019 The American Association of Suicidology doi: 10.1111/sltb.12556		Quantitative	Study 1: 1.006 adults (53% men) Study 2: 190 psychiatryc adults (47% men)
49	Patterson A, Holden R. (2012) Psychache and Suicide Ideation among MenWho AreHomeless: ATest of Shneidman's Model Suicide and Life-Threatening Behavior 42(2) April 2012 147 2012 The American Association of Suicidology doi: 10.1111/j.1943-278X.2011.00078.x	2012	Quantitative	97 men
50	Pereira E, Kroner D, Holden RR, Flamenbaum R. Testing Shneidman's model of suicidality in incarcerated offenders and in undergraduates. Pers Individ Dif (2010) 49:912–7. doi: 10.1016/j.paid.2010.07.029	2010	Quantitative	73 imprisoned men, 80 students men; 80 students women
51	Pompili M, Lester D, Leenars A, Tatarelli R, Girardi P. Psychache and suicide: a preliminary investigation. Suicide Life Threat Behav (2008) 38(1):116–21. doi: 10.1521/suli.2008.38.1.116	2008	Quantitative	88 inpatients (35 men y 53 women)
52	Pompili, M. (2018). The increase of suicide rates: the need for a paradigm shift. thelancet.com Vol 392 August 11, 2018	2018	Revission	Theory revission
53	Reisch T, Seifritz E, Esposito F, Wiest R, Valach L, Michel K. An fMRI study on mental pain and suicidal behavior. J Affect Disord (2010) 126(1):321–5. doi: 10.1016/j.jad.2010.03.005	2010	Mixed	Recent (past month) suicide attempters
54	Rizv. ia S, Iskrica A, Calatic R, & Courtetc P. (2017) Psychological and physical pain as predictors of suicide risk: evidence from clinical and neuroimaging findings. 0951-7367 Copyright 2017 Wolters Kluwer Health, Inc. All rights reserved. doi: www.co-psychiatry.com	2017	Systematic revisión	Systematic revission
55	RuthTrakhtenbrot, YariGvion, YossiLevi-Belz, NettaHoresh, TsviFischel, MarkWeiser, IlanTreves, Alan Apter (2016) Predictive value of psychological characteristics and suicide history on medical lethality of suicide attempts: afollow-upstudy of hospitalized patients. Journal ofAffectiveDisorders199(2016)73–80. doi: http://dx.doi.org/10.1016/j.jad.2016.03.054_0165-0327/ & 2016Elsevier	2016	Quantitative	153 psyquiatric patients
56	Segal-Engelchin, D., Kfir-Levin, N., Neustaedter, S. B., & Mirsky, J. (2015). Mental pain among female suicide attempt survivors in Israel: An exploratory qualitative study. International Journal of Mental Health and Addiction, 13(4), 423–434. doi: https://doi.org/10.1007/s11469-015-9545-2	2015	Qualitative	4 women suicide attempters (21–58 years old)
57	Shelef L, Fruchter E, Hassidim A, Zalsman G. Emotional regulation of mental pain as moderator of suicidal ideation in military settings. Eur Psychiatry (2015) 30:765–9. doi: 10.1016/j.eurpsy.2014.12.004	2015	Quantitative	168 soldiers (100 men: 59,5%) 18–21 years old
58	Schuck, A., Calati, R., Barzilay, S., Bloch-Elkouby, S., Galynker, I (2018). Suicide Crisis Syndrome: A review of supporting evidence for a new suicide-specific diagnosis. Behav Sci Law. 2019;37:223–239. wileyonlinelibrary.com/journal/SBl doi: 10.1002/SBl.2397	2018	Revisión	Bibliographic revission
59	Skevington, S., Lotfy, M. & O'Connell, K. The World Health Organization's WHOQOL-BREF quality of life assessment: Psychometric properties and results of the international field trial. A Report from the WHOQOL Group. Qual Life Res 13, 299–310 (2004). doi: https://doi.org/10.1023/B:QURE.0000018486.91360.00.	2004	Quantitative	11.830 adults from 23 countries
60	Soumani, A., Damigos, D., Oulis, P., Masdrakis, V., Ploumpidis, D., Mavreas, V., & Konstantakopoulos, G. (2011). Mental pain and suicide risk: Application of the greek version of the mental pain and the tolerance of mental pain scale. Psychiatriki, 22(4), 330–340.	2011	Quantitative	112 participants (73 women y 39 men). 18–65 years old
61	Rui C. Campos, Margarida Gomes, Ronald R. Holden, Margarida Piteira & Ana Rainha (2017) Does psychache mediate the relationship between general distress and suicide ideation?, Death Studies, 41:4, 241–245. doi: 10.1080/07481187.2016.1251510	2017	Quantitative	440 students
62	Tossani, E., Ricci Garotti, M. G., Mikulincer, M., Giovagnoli, S., Calzolari, G., Landi, G., & Grandi, S. (2021). Psychometric evaluation of the Italian version of Orbach & Mikulincer mental pain scale in a non-clinical sample. Current Psychology, 40(4), 1903–1910. doi: https://doi.org/10.1007/s12144-019-0128-4	2021	Quantitative	544 italian adults italianos, 18–85 years old
63	Troister T, Davis MP, Lowndes A, Holden RRA. Five-month longitudinal study of psychache and suicide ideation: replication in general and high-risk university students. Suicide Life Threat Behav (2013) 43(6):611–20. doi: 10.1111/sltb.12043	2013	Quantitative	4.499 students (80% women). 17–89 years old
64	Troister T, Holden RR. A two-year prospective study of psychache and its relationship to suicidality among high-rosk undergraduates. J Clin Psychol (2012) 68(9):1019–27. doi: 10.1002/jclp.21869	2012	Quantitative	41 students with suicide risk
65	Troister T, Holden RR. Comparing psychache, depression, and hopelessness in their associations with suicidality: a test of Shneidman's theory of suicide. Pers Individ Dif (2010) 49:689–93. doi: 10.1016/j.paid.2010.06.006	2010	Quantitative	1,475 student, 71% mujeres
66	Troister T, Holden RR. Factorial differentiation among depression, hopelessness, and psychache in statistically predicting suicidality. Meas Eval Couns Dev (2013) 46:50–63. doi: 10.1177/0748175612451744	2013	Quantitative	2.974 students (2.135 women, 769 men, 70 non-declared genre
67	Van Heeringen K, Van den Abbeele D, Vervaet M, Soenen L, Audenaert K. The functional neuroanatomy of mental pain in depression. Psychiatry Res (2010) 181:141–4. doi: 10.1016/j.pscychresns.2009.07.011	2010	Quantitative	39 patients (22 women, 17 men) diagnosed by depression
68	Xie W, Li H, Luo X, Fu R, Ying X, Wang N, et al. Anhedonia and pain avoidance in the suicidal mind: behavioral evidence for motivational manifestations of suicidal ideation in patients with major depressive disorder. J Clin Psychol (2014) 70(7):681–92. doi: 10.1002/jclp.22055	2014	Quantitative	40 outpacients and 20 control
69	Xuemei Sun, Huanhuan Li, Wei Song, Songyuan Jiang, Chengfeng Shen, Xiang Wang (2020). ROC analysis of three-dimensional psychological pain in suicide ideation and suicide attempt among patients with major depressive disorderJ. Clin. Psychol. 2020;76:210–227.doi: 10.1002/jclp.22870	2020	Quantitative	137 pacients (52 men, 38%; 85 women, 62%).
70	Yossi Levi-Belz, Yari Gvion, Alan Apter (2020). The Serious Suicide Attempts Approach for Understanding Suicide: Review of the Psychological Evidence. OMEGA—Journal of Death and Dying 1-18. doi: 10.1177/0030222820981235	2020	Review	Revision
71	You Z, Song J, Wu C, Qin P, Zhou Z. Effects of life satisfaction and psychache on risk for suicidal behaviour: a cross-sectional study based on data from Chinese undergraduates. BMJ Open (2014) 4:e004096. doi: 10.1136/bmjopen-2013-004096	2014	Cross-sectional study	5,988 university students
72	Berlim MT, Mattevi SB, Pavanello DP, Caldieraro MA, Fleck MPA, Wingate LR, et al. Psychache and sucidality in adults mood disordered outpatients in Brazil. Suicide Life Threat Behav (2003) 33(3):242–8. doi: 10.1521/suli.33.3.242.23220	2003	Quantitative	*N* = 60 outpatents, 50 women, 10 men

In the next step of the analysis, the groupings obtained were assembled under concepts of abstraction, as shown below:

- The diagnostic variables.
- The psychological experience variables.
- The trait variables.

Grouping by similarity allowed us to guide the clinical reflection of these maladaptive ways of being with oneself and with others in order to select pertinent psychotherapeutic interventions for these conditions that are associated with suicidal risk. We discuss the clinical recommendations in the final section.

To visualise the words that emerged in the analysis, they were organised in proportion to the number of times they appeared in the revised texts. We extracted those words that appeared at least 10 times and presented them in word clouds. This criterion had the simple function of ensuring that the words were visible in the graph, as words with lower frequencies would be too small to be clearly seen.

For the diagramming, we used the free online program EdWordle, a tool that organises word clouds based on Wordle using concepts or groupings of words. The result graphically shows what is presented in a text according to the weight of the words as a whole (http://www.edwordle.net/).

**Figure 1:**
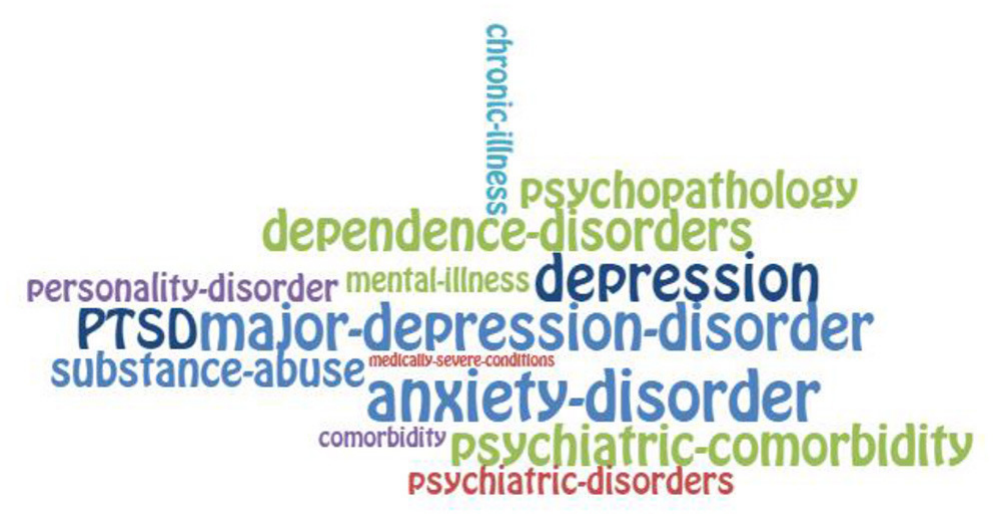
State of suicidal mental pain: Diagnosis.

**Figure 2:**
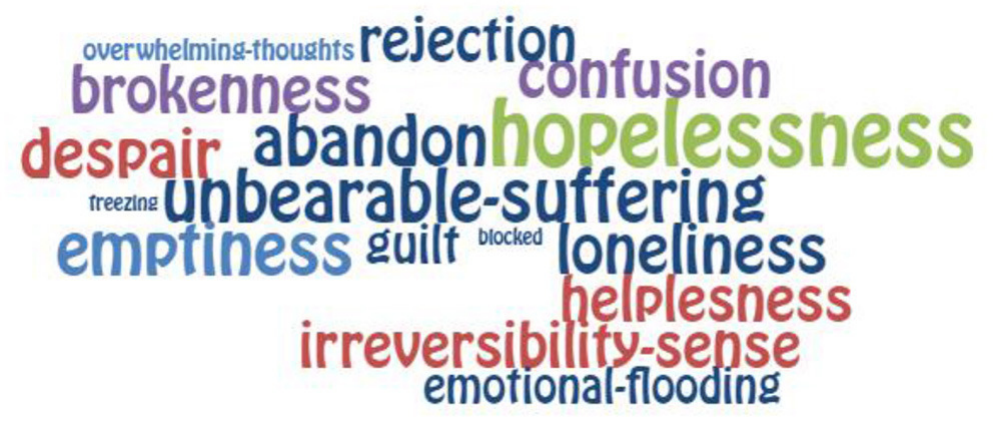
State of suicidal mental pain: Psychological experience.

**Figure 3:**
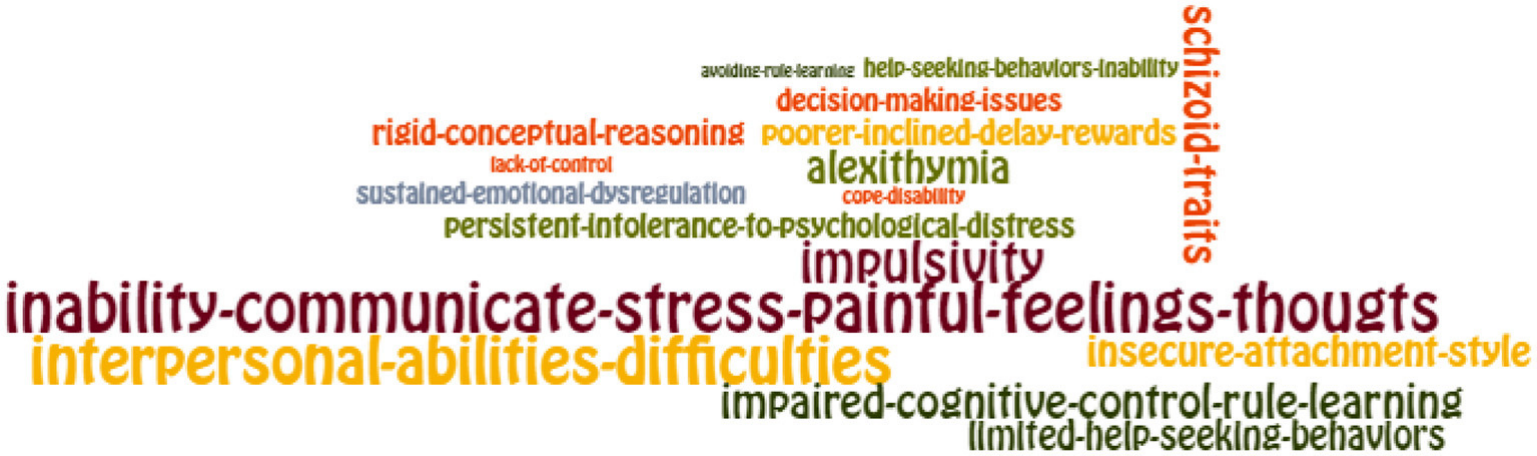
Traits of suicidal mental pain.

The layout can be seen in Figures 1, 2 “Groupings of concepts of state in mental pain (diagnosis and psychological experience, respectively)” and in Figure 3 “Groupings of concepts of traits in mental pain.”

Finally, in the discussion, we propose strategies to use these findings in such a way that they serve as a response to difficult circumstances, interpersonal adversities, and psychological suffering that seems inevitable and is intolerable. This can be oriented toward aspects such as

**Figure 4:**
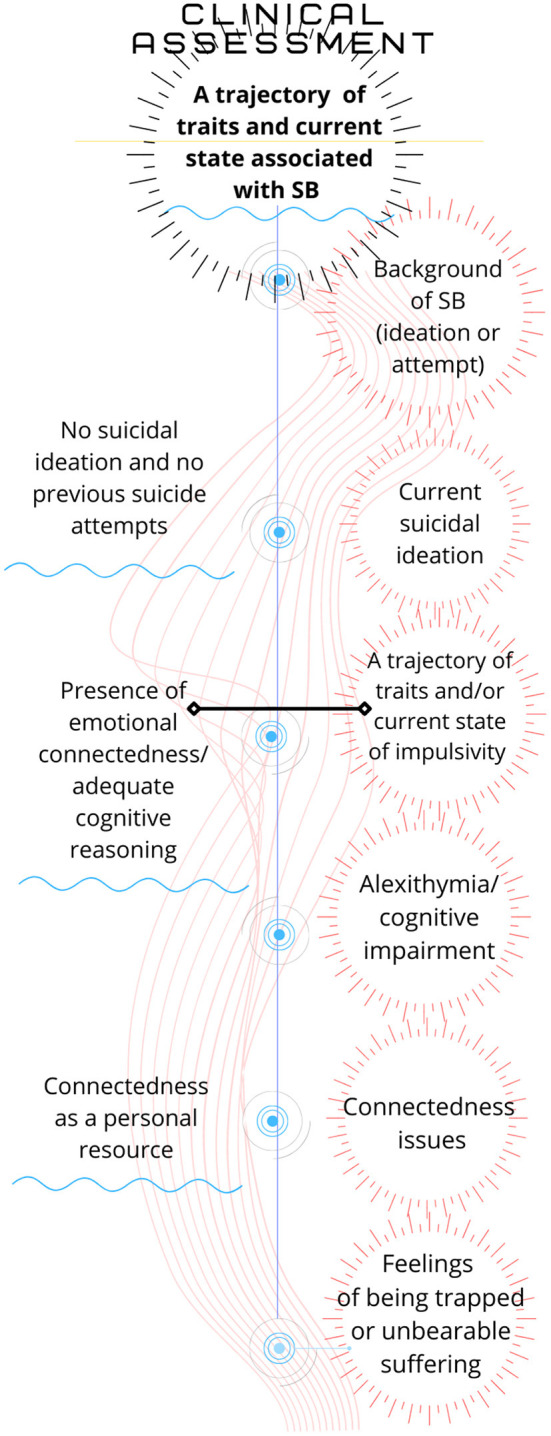
Clinical assessment.

1) the clinical interview in the evaluation of patients with probable risk of suicide and

2) therapeutic interventions in different stages of the treatment of subjects with suicidal risk from the first clinical interview onwards (Figure 4).

## Results

The 73 studies reviewed had different characteristics, amongst which the following stand out:

The works included different methods and approaches. Most of them were done in adult samples. Not all studies detailed the sex and age of the patients, and some that obtained information from the internet were unable to establish the samples' demographic characteristics. There were 56 studies with quantitative methods, most of them comparing the results between different samples; 4 studies used qualitative analysis with smaller samples, and 2 studies used mixed methods. There were 4 reviews, 3 systematic reviews, 1 cross-sectional survey, and 2 studies that followed different methods. There were 2 studies in which a follow-up of close to 1 year was carried out. While most of the studies came from the United States, there were three Portuguese studies, two Canadian, and four Chinese. The reviewed studies are detailed in Table 1.

We analysed the information through an abstraction exercise that allowed us to classify different items in ordered groups under a common notion. The content analysis was structured based on the personality theory perspective of states and traits in suicidal mental pain. There are different factors that shape personality and can lead to dysfunctionalities in coping with areas of life: the genetic load that has been inherited from family biology; learning to deal problematically with emotions and interpersonal relationships in childhood; life events that cause prolonged stress; and traumatic childhood experiences, such as abuse, neglect, loss of caregivers, and bullying, among others. Dysfunctionalities can be increased or decreased, depending on history and context, leading to states of greater or lesser emotional well-being and impacting the relationship with oneself, interpersonal relationships, and quality of life (30).

The analysis provided a list of 607 variables, which an exercise of conceptual abstraction led us to group according to clinical criteria, differentiating those that are transitory from those that are relatively stable over time. The transient variables include the mental pain psychological states in suicide, and relatively stable variables include the mental pain psychological traits in suicide.

### Mental Pain Psychological States in Suicide

1) Psychological states associated with a diagnosis of mental pain appearing at least 10 times in the analyses, resulting in 113 concepts. The cut-off point of 10 occurrences was chosen purely for the purpose of visibility in the diagramming of the concepts found. The diagnostic variables found were the following: Anxiety disorder, Chronic Illness, Comorbidity, Dependence disorders, Major depression, Medically severe conditions, Mental Illness, Personality disorder, Psychiatric comorbidity, Psychiatric disorders, Psychopathology, Post-traumatic stress disorder (PTSD), and Substance abuse. These factors were abstracted into the grouping concept called Pathology.

2) Psychological states associated with a psychological experience of mental pain appearing at least 10 times in the analyses, resulting in 222 concepts. The psychological experience state variables found were as follows: Abandon, Blocked Sense, Brokenness, Confusion, Despair, Emotional Flooding, Emptiness, Freezing, Guilt, Helplessness, Hopelessness, Irreversibility Sense, Loneliness, Overwhelming Thoughts, Rejection, and Unbearable Suffering. These factors were abstracted under the grouping concept known as Unbearable Psychological Suffering.

### Mental Pain Psychological Traits in Suicide

3) Psychological traits of mental pain appearing at least 10 times in the analysis, appearing 272 concepts. The psychological traits variables found were Alexithymia, Inability to Communicate Stress/ Painful Feelings/Thoughts, Lack of Control, Persistent Intolerance of Psychological Distress, and Sustained Emotional Dysregulation. These factors were abstracted under the grouping concept known as Emotional Dysregulation and Impairment in Recognising and Communicating Emotions.

Besides, the results showed other traits as Avoiding Rule-Learning, Inability in Help-Seeking Behaviours, Insecure Attachment Style, Difficulties with Interpersonal Abilities, Limited Help-Seeking Behaviours, and Schizoid Traits. These were abstracted under the grouping concept Troubled Interpersonal Relationships.

Other appearing traits were Impulsivity and Poorer Inclined Delay Rewards, which were named Impulsiveness. Also, we found Coping with Disability, Decision-Making Issues, Impaired Cognitive Control Rule Learning, and Rigid Conceptual Reasoning. These factors were abstracted under the concept Distorted Reasoning.

Both psychological states (transient) and subjective styles of experience (relatively stable personality) can be modified by psychotherapeutic work oriented toward aspects such as resignification of life history and broadening of coping styles.

These variables can be observed in Table 2, States in Mental Pain, and Table 3, Traits in Mental Pain.

**Table 2:** Psychological states (transient) obtained in concepts analysis.

**State variables**								
**Diagnosis**	**Quantity**	**Percentage**	**Concepts abstraction**	**Psychological experience**	**Quantity**	**Percentage**	**Concepts abstraction**
Anxiety disorder	18	15.93	**Pathology**	Abandonment	17	7.66	**Unbearable**
Chronic illness	4	3.54		Blocked	5	2.25	**Psychological**
Comorbidity	3	2.65		Brokenness	15	6.76	**suffering**
Dependence disorders	10	8.85		Confusion	15	6.76	
Depression	15	13.27		Despair	16	7.21	
Major depression disorder	14	12.39		Emotional flooding	10	4.50	
Medically severe conditions	2	1.77		Emptiness	18	8.11	
Mental illness	4	354		Freezing	5	2.25	
Personality disorder	5	4.42		Guilt	11	4.95	
Psychiatric comorbidity	10	8.85		Helplessness	14	6.31	
Psychiatric disorders	5	4.42		Hopelessness	28	12.61	
Psychopathology	8	7.08		Irreversibility sense	14	6.31	
PTSD	9	7.96		Loneliness	16	7.21	
Substance abuse	6	5.31		Overwhelming thoughts	6	2.70	
				Rejection	14	6.31	
				Unbearable suffering	18	8.11	
**Total**	**113**	**100.00**		**Total**	**222**	**100.00**	

**Table 3:** Subjective styles of experience (relatively stable personality) obtained in concepts analysis.

**Trait variables in mental pain**			
			**Concepts**
**Trait factors**	**Quantity**	**Percentage**	**Abstraction**
Alexithymia	17	6.25	Emotional
Inability to communicate, stress, painful feelings/thoughts	42	15.44	dysregulation and impairment in recognising and
Lack of control	7	2.57	communicating
Persistent intolerance of psychological distress	11	4.04	emotions
Sustained emotional dysregulation	9	3.31	
Avoiding rule-learning	6	2.21	Troubled
Help-seeking behaviours inability	8	2.94	interpersonal relationships
Insecure attachment style	16	5.88	
Difficulties with interpersonal abilities	38	13.97	
Limited help-seeking behaviours	14	5.15	
Schizoid traits	18	6.62	
Impulsivity	26	9.56	Impulsiveness
Poorer inclined delay rewards	12	4.41	
Cope disability	7	2.57	Distorted reasoning
Decision-making issues	10	3.68	
Impaired cognitive control rule learning	19	6.99	
Rigid conceptual reasoning	12	4.41	
	272	100	

For a graphic look at the weight of the concepts according to the number of repetitions that were observed in the analysis, we can observe (Figures 1–3).

Psychotherapeutic intervention in these cases can be developed with reflective exercises that add life history accounts and by encouraging adaptive responses to adverse events.

The ability to learn, to modify behaviour from new experiences, explains the usefulness of certain therapeutic interventions to modify the way of understanding and acting in response to various emotional stimuli (31). In the discussion, we will review some clinical interventions that would make it possible to reduce MP by expanding the repertoire of emotional and behavioural responses of a subject at risk of suicide (Figure 3).

## Discussion

The purpose of this paper is to contribute to the accuracy of the understanding of MP in suicide risk. Through a qualitative review of content, we have found variables which can be grouped corresponding to clinical criteria, differentiating between transitory variables associated with a psychological state in suicidal MP, including diagnostic variables on the one hand and subjective experience variables on the other.

In addition, we found relatively stable personality traits in suicidal MP. Figure 3 shows the central elements of the evaluation of a patient who might be suffering from MP. Each of the factors is a reminder of the facts that are paramount in this task. We have collected the results of a number of studies to highlight those aspects that are most frequently observed in a clinical population. These facts can be assessed in the first interview and at different stages of the treatment after a suicide attempt. Our recommendations are based on the identification of mental pain as the event closest to suicidal action. One of the limitations of our study on SB is that the studies selected are those that include MP, which leaves out many very valuable papers conducted from different perspectives. We understand that the problem of suicide is extraordinarily complex, and therefore, when evaluating the experience of mental pain, we can recognise the manifestation of a diversity of facts in a similar state.

The main task for clinicians is to identify what contributes to encouraging or attenuating psychic pain in the subject's relationships with him/herself and with others. To reduce psychic pain, interventions must be directed at those factors that determine MP in everyone. The treatment of axis I disorders (DSM-V) is a priority and has been sufficiently described for each of the pathologies by means of various clinical guidelines and systematic reviews. We will review other state variables, such as hopelessness, unbearable suffering, loneliness, and psychological pain, when describing clinical strategies and the proper psychotherapeutic interventions.

The different groups of factors associated with suicide—namely personality and individual differences, cognitive factors, social factors, and adverse life events (1)—play roles in the different stages of this behaviour. According to the integrated motivational-volitional model proposed by O'Connor, SB has a pre-motivational phase that includes background factors and triggering events: diathesis, environment, and life events; a motivational phase, in which ideation and intention build up; and a volitional phase, where the behaviour is enacted. MP takes place in the motivational phase, in which diverse psychological factors might lead a subject to experience a feeling of humiliation and defeat, followed by a perception of entrapment that might lead him/her to conceive of SB as the only way to alleviate mental pain. It is in the motivational phase where the presence or absence of factors might show a risk or a protective role. It is within that phase—which has a limited time frame—that those interventions might play a critical role in helping someone to avoid SB. The myriad of possible factors that might be relevant to someone's actions obliges us to select the most relevant ones leading to MP in each individual case. There doesn't seem to be a better way to approach this clinical plight. To prevent the subject from acting, we must single out the sources of individual psychological distress, addressing individual needs to foster preventive strategies (32).

### State of Unbearable Psychological Suffering and Feelings of Hopelessness and Loneliness

In historical periods when the population has been exposed to unusual suffering under circumstances of great uncertainty and real threat, suicide has increased significantly. This is what happened in some parts of Germany after the end of World War II (33). But exceptional historical circumstances are not sufficient to explain what is eminently, rather, a matter of subjective experience. A sentimental break-up, an economic failure, and a severe illness are experiences associated with different responses. Each person finds his or her own way of adapting to adversity.

Unbearable suffering, along with depression, hopelessness, and impulsivity, is one of the most frequent emotional experiences in MP (34). This is consistent with our findings (Figure 1). A comprehensive model sustains that SB occurs in the presence of four factors: psychological pain, hopelessness, a feeling of connectedness, and suicide capacity. As summarised by Klonsky, “we believe that any effort to prevent or treat suicidality should target one or more of these four factors and will succeed to the extent that one or more of these factors is changed for the better” (34). Moreover, “connectedness is most relevant to suicidal ideation as a protective factor among those high on pain and hopelessness, especially when one's connectedness exceeds one's pain” (34) Hence, pain, hopelessness, and connectedness contribute to SB interactively and jointly.

Our data support the joint role that these factors have in the experience of mental pain. The presence of unbearable pain and hopelessness (in Figure 1) has a similar relevance to “interpersonal difficulties” and “inability to communicate thoughts feelings” (Figure 2). Both capacities are expressed in the ease or difficulty of dealing with interpersonal differences and maintaining the bond with others (connectedness). There is a need to protect and activate cognitive and emotional resources using psychological interventions to foster a sense of emotional connexion with the people available in the patient's environment. In a suicidal crisis, patients may not visualise who could be part of their network. Nonetheless, this critical intervention can prevent suicidal ideation from escalating in intensity for those who are experiencing both pain and hopelessness. Also, when hopelessness is present, these interventions are often useful even if the patient is not depressed (35).

### Emotional Dysregulation and Impairment in Recognising and Communicating Emotions

Alexithymia, defined as the inability to identify or express emotions (36), has been associated with SB. A recent systematic review and meta-analysis of the association between alexithymia and SB “found a large size effect (0.54) in the meta-correlation of alexithymia and suicide ideation, and a medium effect size (0.25) in the meta-correlation between alexithymia and SB” (37). Similarly, our review of the most frequent terms used in discussing mental pain highlights alexithymia as a trait that might interfere with the capacity to recognise the sources of mental pain. In such cases, when the pain becomes unbearable, the confusion and emotional overflow can be understood as a call for help and support (38, 39).

In the previous stages of treatment, it is necessary to do the task of naming the emotions that are usually expressed as physical sensations and emotional storms. This exercise of cognitive structuring allows the increase of emotional awareness, with the development of progressively more sophisticated abilities to differentiate and integrate the contents of emotional activity” (40).

Mentalisation treatment is helpful to understand mental states, such as beliefs, emotions, intentions, and desires, which are the basis of the relationships with oneself and others. The development of this skill is stimulated by the repetitive exercise of asking how the patient and the others may have felt in an interaction and what they thought and what they intended in a determined situation (41).

### Troubled Interpersonal Relationships

Suicide attempters often have repeatedly experienced frustrated psychic needs in interpersonal interactions, with significantly increased victimisation in their historical contexts, including recurrent situations of physical and psychological abuse. These adverse events often interfere with the development of stable and harmonious interpersonal bonds, as the patients' processes of interpersonal trust, openness, and bonding in relationships have been traumatised (42). Depressive symptoms, such as psychache, thwarted belongingness, and perceived burdensomeness, are also related to suicidality through intrapersonal and interpersonal variables (43).

Clinical work can be guided by stimulating the patient's abilities to understand the context of the emotions arising in relationships (both one's own and those of others); to direct his/her attention to what is relevant for problem-solving; to adopt different points of view and multiple perspectives on issues; to discriminate the importance of events; to make his/her expectations flexible; and to concede and ask for changes to find agreed solutions (44). This ability can be obtained through joint reflection on different points of view and expectations. In addition, what the patient expects to happen and reflections on what he/she imagines others expect allow for greater flexibility in those particularly rigid views the subject may have about himself and others.

### Impulsivity in Distorted Reasoning

Impulsivity has always been considered to play a major role in SB. However, it is difficult to determine its actual role in SB. A particularly complex task is to figure out its place within the sequence of events preceding suicide. For several authors, impulsivity is critical to explaining these actions. According to a recent study based on the Interpersonal Theory of suicide, “trait impulsivity is related to suicidal behaviour and the fluctuation of suicidal ideation, but not to suicidal ideation itself. Thus, trait impulsivity seems to act as a distal risk factor via capability for suicide and it seems to play a role for the dynamics of suicidal ideation” (45). However, impulsivity can manifest itself in different stages of behaviours leading to suicide, from the moment in which the agent evaluates his or her difficulties and considers the options to solve them until the moment in which he or she decides to act. As we said before, SB is the result of a behaviour marked by ambivalence in which an individual conceives of actions without knowing the true consequences they would have for him/herself as well as for others. Not having the possibility of assessing the consequences of one action on another, the agent, motivated by the need to end his/her anguish and overwhelmed by the hopelessness of not finding a way out of his/her difficulties, will try to end his/her life. The action will occur under the motivational pre-eminence of one or several factors. A decision with irreversible consequences supported by a narrow view of the circumstances and encouraged by a state of emotional restraint almost always originates in an impulsive thought or behaviour. SB can be understood as acts that occur—in the presence of poor decision-making and impulsivity—when someone experiences unbearable suffering. This emotional state has been recognised as a condition that might stimulate someone to take their own life as a solution to the misery they are experiencing (46, 47).

In the clinical evaluation of patients at risk of SB, clinicians should take into consideration the patient's overall situation, avoiding a judgement based solely on a single aspect of it (Figure 4). Impulsivity might be a trait in someone's personal history, and yet, they may not have a history of SB. On the other hand, some patients may not have the trait of impulsivity in their personal history, but they might be suffering from some clinical conditions, such as a mixed bipolar state or psychosis, which by themselves increase the risk of decisions based on the patient's acute emotional status at a particular moment.

Highly lethal SB has been found in the presence of an overwhelming sense of entrapment with cognitive and emotional dysregulation (6). High-lethality suicide attempters, independent of depressive symptoms, may present executive function impairments, such as rigidity-shift setting and negative cognitive biases (48).

Suicidal individuals are more likely to inaccurately interpret stressors, use emotion-focused coping, and adopt an avoidant approach. In such cases, we must prioritise the application of techniques such as cognitive remediation and problem-solving (49, 50). Problem-solving efforts are multifaceted (social, psychological) and encompass a wide range of cognitive and emotional functions, such as autonomy, mastery of the environment, and development of a meaningful purpose in life (51).

Cognitive techniques aimed at reformulating and resolving intrapersonal and interpersonal conflicts are of great help for the subject to find adequate alternatives to understand and act whilst avoiding the feeling of entrapment. To do this, it will be necessary to look at each of the cognitive dimensions on which the most complex judgments are based. Among them are attention, memory, executive function, metacognition, and attribution of intentions. The goal of these interventions is to stabilise and generalise both intrapersonal and interpersonal functioning (52–64).

The study of suicidal behaviour is fraught with difficulties and limitations. In fact, it faces the same limitations that we find in the understanding of human behaviour and, particularly, of intention. An intentional action is one that someone performs for some reason. Human behaviour can have similar consequences for different reasons. But it is not possible to understand the reasons that led someone to act based solely on the consequences of their actions (65–73).

It is possible that in those subjects who are in treatment for mental health problems, as well as in those who have a history of previous suicide attempts, MP is the state closest to suicidal action. Therefore, if we concentrate our efforts on this group of patients, we may be able to intervene and prevent what would otherwise be inevitable (71, 74–80). We must be realistic that this is a subgroup of the total number of suicides. But it is precisely this group that is usually most within reach of our treatment.

The above clinical recommendations to address the state and trait aspects of the individual experience of MP can give clinicians (psychologists and psychiatrists) the appropriate means to alleviate psychological distress and prevent suicidal risk.

A limitation of the research is that it focused only on studies presented in English, without including scientific articles in other languages. Therefore, further research that expands the vocabulary may contribute to enriching the explanation of the subjective experience of suicidal behaviour. Likewise, it would be desirable to study the interventions proposed for states and traits in SB and to deepen the knowledge of the impact they have on decreasing the psychological distress of suicidal risk.

## Author Contributions

All authors listed have made a substantial, direct, and intellectual contribution to the work and approved it for publication.

## Funding

This work was funded by the Department of Psychiatry of the School of Medicine at the Pontificia Universidad Católica de Chile. The study received funding from ANID – Millennium Science Initiative Program/Millennium Institute for Research on Depression and Personality (MIDAP) ICS13_005.

## Conflict of Interest

The authors declare that the research was conducted in the absence of any commercial or financial relationships that could be construed as a potential conflict of interest.

## Publisher's Note

All claims expressed in this article are solely those of the authors and do not necessarily represent those of their affiliated organizations, or those of the publisher, the editors and the reviewers. Any product that may be evaluated in this article, or claim that may be made by its manufacturer, is not guaranteed or endorsed by the publisher.
